# Fibromatosis colli or pseudotumour of sternocleidomastoid muscle, a rare infantile neck swelling

**DOI:** 10.1016/j.bjorl.2020.12.003

**Published:** 2021-01-02

**Authors:** Nasser Alrashidi

**Affiliations:** Qassim University, Unaizah College of Medicine and Medical Sciences, Department of Surgery, Al-Qassim, Saudi Arabia

## Introduction

Fibromatosis colli is a rare benign disease of the lower part of the sternocleidomastoid (SCM) muscle, also known as infancy sternocleidomastoid pseudotumor. The term “tumor” is misleading as it will not be a cancerous disease but is referred to as a congenital fibrotic phase, so the word tumor means swelling in this particular sense.^1^

This disease has an incidence of 0.4% in babies. In 75% of cases, it is generally unilateral, affects the right side, and male patients significantly higher frequencies than female patients. Complicated delivery history and birth injury may be associated in more than 50% of cases.^2^

Using ultrasound (USG), fibromatosis colli can be accurately diagnosed, thereby excluding more suspicious swellings of the neck and face. Proper diagnosis of ultrasound may prevent inappropriate investigation and medical interventions which could be harmful.^3^

We report a case study in which clinical examination and ultrasound in an infant revealed the diagnosis of fibromatosis colli.

## Case presentation

**Figure 1:**
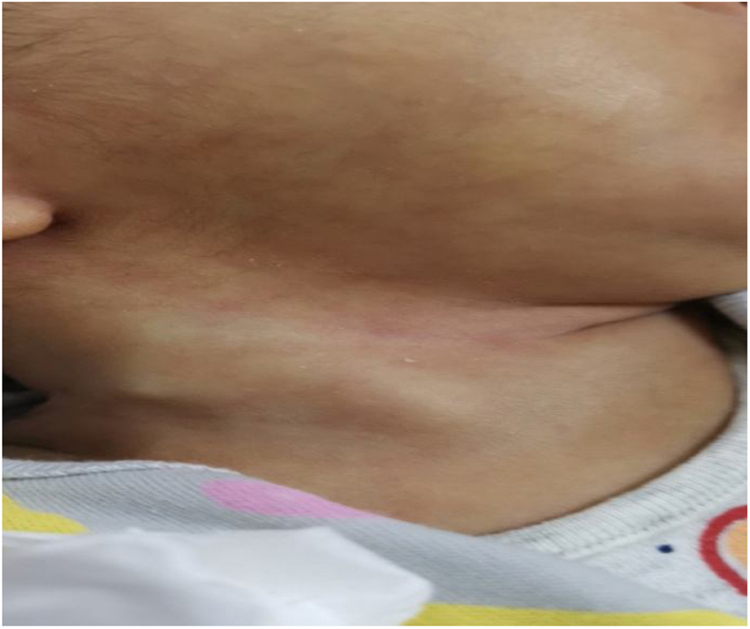
Right neck swelling.

A 4 week old girl, full-term, with normal spontaneous vaginal delivery, was born to multiparous mother with 3 kg birth weight, and referred to the clinic with right-sided neck swelling since 2 weeks of age. According to the mother no obstructive symptoms or difficulty in oral feeding existed. Examination revealed an active baby with a right-sided neck mass around 2–3 cm over level 2 and level 3, firm in consistency, mobile within the SCM muscle, not pulsatile and with no signs of infection (Fig. 1). There was a limited range of motion upon rotation of the neck to the side. Other physical examinations were normal. Laboratory investigations were normal. Hence, the neck ultrasound was done to clarify her presentation.

**Figure 2:**
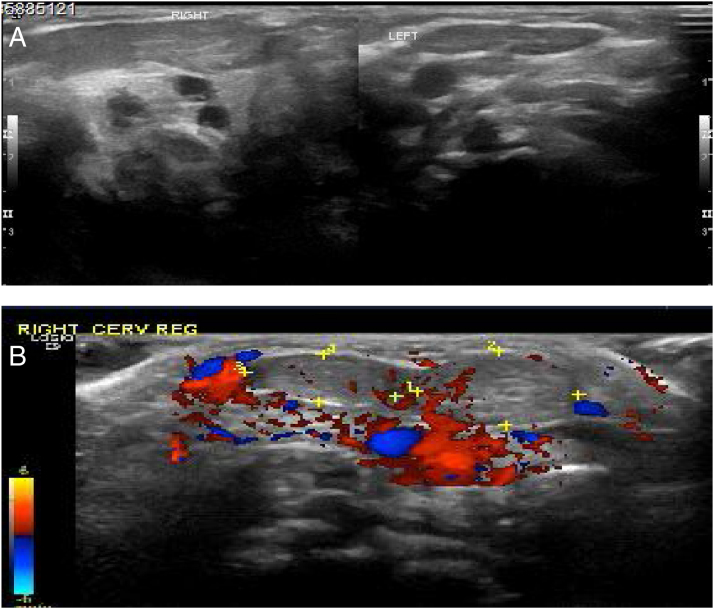
The ultrasound finding on figure A and B demonstrate bulky right sternocleidomastoid muscle as compared to the left side.

The USG showed the right SCM was diffusely enlarged and thickened involving the muscle belly, giving a fusiform/ellipsoid appearance measuring around 2.5, 2.4 and 1 cm. The contralateral neck appeared normal. There was no abnormal internal vascularity or internal hyperechoic/calcify foci. A few mildly prominent bilateral cervical lymph nodes were noted. The thyroid and salivary glands were normal (Fig. 2). Based on the clinical and radiological assessments, a fibromatosis colli as the diagnosis was made. The parents were instructed about the details of physiotherapy. Upon followup assessment, there is a progressive reduction in the size of the mass until complete disappearance after the age of 3 months.

## Discussion

While the pathophysiology of fibromatosis colli is not understood, it is possibly linked to the birth trauma of delivery forceps, breech presentation, prolonged hard labor, and primiparous birth. The obstructed venous outflow in the muscle is one of the most likely explanations, either during delivery or during intrauterine growth. The injury causes necrosis and then fibrosis in the muscle fibers, resulting in the production of secondary muscle strain. Another hypothesis proposes that the lesion results from a selective injury to the SCM muscle in the in utero fetal head position. Such injury results in secondary compartment syndrome and consequent pressure necrosis and fibrosis within the muscle.^1^, ^4^

Neck swelling during infancy and particularly through the first weeks of life is a generally indicative clinical sign of fibromatosis colli. Clinically, abscesses, lymph nodes, thyroid lobe dislodging, and bronchogenic cysts are readily differentiated from fibromatosis as well as from cancerous lesions such as lymphoma, teratoma, fibrosarcoma, and rhabdomyosarcoma, as well as benign neoplastic disorders such as cystic hygroma and hemangioma. Vascular malformation (particularly lymphatic malformation/lymphangioma) is also to be included in the differential diagnosis, in addition to bronchogenic cysts and thyroglossal duct cysts. Abscess is less likely in a small baby but more possibly in an immunodeficiency environment, or in older babies. Although fibromatosis colli is a pathology that theoretically could result in respiratory failure and ultimately tracheostomy, the literature has never recorded such evidence.^1^, ^2^, ^4^, ^5^

USG is the best diagnostic tool since it is fast, non-invasive, and safe. USG with Doppler can be used to describe the waveform with high resistance. Simultaneous motion between the mass and the remainder SCM is seen in real-time sonography.^6^ This also is identified in a computed tomography (CT) scan and magnetic resonance image (MRI). The sternocleidomastoid muscle appears diffusely swollen, attenuated to isodense on CT scan. Due to the presence of fibrous tissue, MRI characteristics include reduced mass signal strength on T2W images relative to gradient recalled T1W images.^7^ The fine needle aspiration cytology can be used but due to similar histological features, it may be mistaken for fibrous neoplasia.^5^

In general, the condition is self-limited. Most of the cases are managed with conservative management. It is expected to resolve itself over a 4–8-month period. It is treated in a non-surgical manner when diagnosed early, which requires passive stretching and physiotherapy.^4^ Surgical intervention may only be needed in refractory cases or occasionally in a child over age one. Different surgical methods include SCM excision, the bipolar release of SCM with Z-plasty muscle bulk reconstruction, and tenotomy or release of the SCM.^4^ The prognosis of children diagnosed and treated when they are older than a year is worse.^8^

Such affected patients need treatment to avoid torticollis-like complications. The twisted neck position can result in positional plagiocephaly and facial asymmetry.^2^, ^8^

## Conclusion

Fibromatosis colli is a rare cause of neck swelling in neonates and infants, in whom ultrasound is the diagnostic method of choice and conservative management may be the only treatment indicated. It is not possible to overemphasize the importance of diagnosing this disorder and thereby prevent more unnecessary evaluations and inappropriate therapeutic interventions.

## Conflicts of interest

The author declares no conflicts of interest.
